# 

**Published:** 1885-01

**Authors:** 


					﻿Vol. VI.	JANUARY, 1885.	No. 1.

I TndnjnuIcntPraclitioncr
A lOKTHLY
DEVOTED TO
DENTAL AND ORAL SCIENCE.
W. C. BARRETT, M. D„ D. D. S.,
EIDITOIR.
The New York Dental Journal Association,
PUBLISHERS,
No. 35 West <fcβth Street, New Yorls,
AND
11 AV. Chippewa St., Buffalo, N. Y.
$2.50 Per Annum in Advance, .... Single Copies, 25 Cents.
Entered at the Post-Office, Buffalo, N. Y., as Second-Class matter.
				

